# Inhibiting ERK Activation with CI-1040 Leads to Compensatory Upregulation of Alternate MAPKs and Plasminogen Activator Inhibitor-1 following Subtotal Nephrectomy with No Impact on Kidney Fibrosis

**DOI:** 10.1371/journal.pone.0137321

**Published:** 2015-09-28

**Authors:** Faith Hannah Nutter, John L. Haylor, Arif Khwaja

**Affiliations:** 1 Academic Unit of Nephrology, Department of Infection and Immunity, Medical School, University of Sheffield, Sheffield, England; 2 Sheffield Kidney Institute, Northern General Hospital, Sheffield, England; UCL Institute of Child Health, UNITED KINGDOM

## Abstract

Extracellular-signal regulated kinase (ERK) activation by MEK plays a key role in many of the cellular processes that underlie progressive kidney fibrosis including cell proliferation, apoptosis and transforming growth factor β1-mediated epithelial to mesenchymal transition. We therefore assessed the therapeutic impact of ERK1/2 inhibition using a MEK inhibitor in the rat 5/6 subtotal nephrectomy (SNx) model of kidney fibrosis. There was a twentyfold upregulation in phospho-ERK1/2 expression in the kidney after SNx in Male Wistar rats. Rats undergoing SNx became hypertensive, proteinuric and developed progressive kidney failure with reduced creatinine clearance. Treatment with the MEK inhibitor, CI-1040 abolished phospho- ERK1/2 expression in kidney tissue and prevented phospho-ERK1/2 expression in peripheral lymphocytes during the entire course of therapy. CI-1040 had no impact on creatinine clearance, proteinuria, glomerular and tubular fibrosis, and α-smooth muscle actin expression. However, inhibition of ERK1/2 activation led to significant compensatory upregulation of the MAP kinases, p38 and JNK in kidney tissue. CI-1040 also increased the expression of plasminogen activator inhibitor-1 (PAI-1), a key inhibitor of plasmin-dependent matrix metalloproteinases. Thus inhibition of ERK1/2 activation has no therapeutic effect on kidney fibrosis in SNx possibly due to increased compensatory activation of the p38 and JNK signalling pathways with subsequent upregulation of PAI-1.

## Introduction

Irrespective of the underlying insult, progressive chronic kidney disease (CKD) is characterised by glomerulosclerosis, tubulointerstitial fibrosis, tubular atrophy and capillary loss. The cellular mechanisms responsible for these histological changes are characterised by infiltration of inflammatory cells, release of fibrogenic growth factors, tubular epithelial to mesenchymal transition (EMT), activation and proliferation of fibroblasts with subsequent accumulation of extracellular matrix (ECM) [1, 2]. Myofibroblasts are believed to be the principal effector cells in fibrogenesis, with increased proliferation of myofibroblasts preceding ECM expansion [3]. Inhibiting excessive myofibroblast proliferation has been shown to reduce fibrosis and may improve kidney function *in vivo [4, 5]*.

The mitogen-activated protein kinases (MAPKs) are a family of serine/threonine kinases that regulate many cellular processes central to kidney fibrogenesis including cell proliferation, apoptosis, EMT and ECM deposition [6]. The three best characterised members of the family, extracellular signal-regulated kinase1/2 (ERK1/2), c-Jun N-terminal kinase (JNK) and p38MAPK all appear to be activated in response to renal injury and contribute to the fibrotic response [6]. ERK1/2 is activated by the Ras-Raf-Mek-ERK signalling pathway as a result of cell-surface receptor activation by mitogenic growth factors. Other regulators of Erk1/2 activity include the tumour suppressor phosphatase and tensin homolog (PTEN) which appears to target Erk1/2 activity by directly regulating Raf/Mek as well as the PI3/AKt signalling cascade [7]. Growth factors may also activate Erk1/2 via phospholipase-gamma(PLC- gamma) [8] and protein kinaseC (PKC) pathways whilst integrins can also regulate the Ras-Raf-MekErk pathways [9]. Furthermore a variety of scaffold proteins including KSR1/2, IQGAP1, MP1, β-Arrestin1/2 have been shown to regulate Erk1/2 activity [10].

Once phosphorylated, ERK1/2 can activate a variety of cytoplasmic proteins as well as translocate to the nucleus where it is involved in the upregulation of an array of transcription factors involved in cell survival and proliferation. In contrast JNK and p38 MAPKs are predominantly responsive to stress stimuli [11]. There is increasing evidence that ERK1/2 is a key mediator of kidney fibrogenesis. For example the induction of EMT in tubular epithelial cells by pro-fibrotic growth factors such as angiotensin II, aldosterone, and transforming growth factor beta-1 (TGF-β1) are mediated by ERK signaling [12–14]. Similarly the production of fibronectin and other ECM proteins by mesangial cells and renal tubular cells in response to stimuli such as high glucose and stretch also appear to be dependent on ERK activation [15, 16]. Furthermore inhibition of ERK1/2 signalling has been shown to reduce cystogenesis in rat model of polycystic disease [17] whilst inhibition of Ras-GTPase (a key upstream regulator of ERK1/2) attenuates fibrosis in a model of folic acid-induced nephropathy [18].

In a biopsy series of human glomerulopathies, immunostaining for phospho-ERK correlated with cell proliferation, tubulointerstitial fibrosis and an increase in alpha smooth muscle actin (α-SMA) positive myofibroblasts [19]. Taken together the experimental and biopsy data suggest that ERK1/2 activation may play a key role in the pathogenesis of kidney fibrosis by pleiotropic effects on cell proliferation, EMT and ECM accumulation [6]. We therefore tested the hypothesis that inhibiting ERK1/2 activation, by targeting its upstream activator MEK, could attenuate experimental kidney fibrosis. CI-1040 is a well-characterised MEK inhibitor that binds to a hydrophobic pocket in MEK, inducing conformational change that locks it into a closed, catalytically inactive form [20]. CI-1040 has entered early clinical trials and has been shown to inhibit cell proliferation *in vivo* in a variety of oncogenic models as well as improving kidney function and attenuating fibrosis in a model of chronic allograft nephropathy [21, 22].

We examined the effects of inhibiting ERK1/2 activation with CI-1040 in the 5/6 subtotal nephrectomy (SNx) model, which is a well-characterised model of progressive kidney fibrosis and excretory kidney failure [23].

## Results

### CI-1040 inhibits ERK1/2 activation but not proliferation in rat fibroblasts

**Fig 1:**
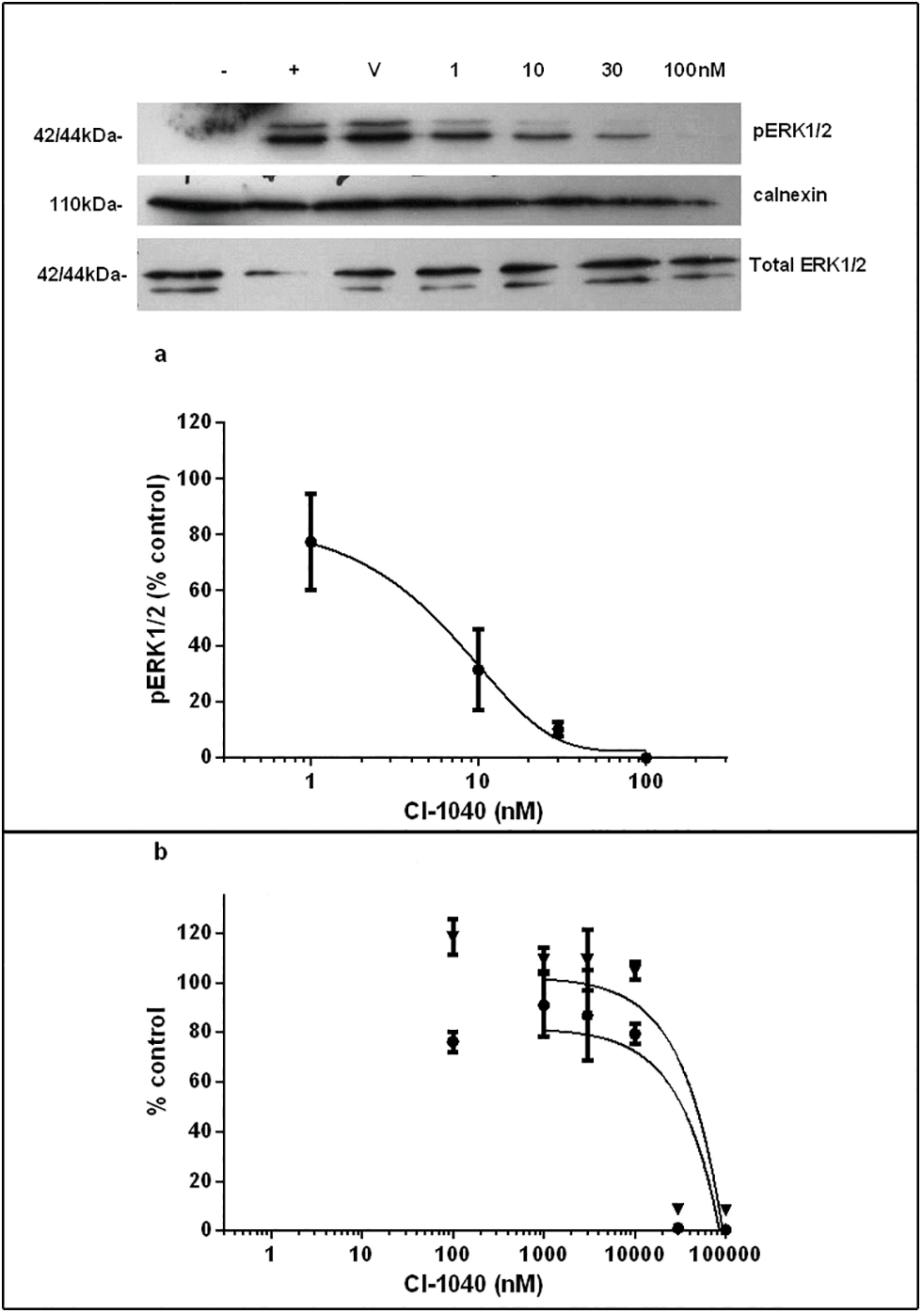
CI-1040 inhibits pERK1/2 activation and proliferation in rat fibroblasts. NRK49F cells were serum starved overnight together with increasing concentrations of the MEK inhibitor CI-1040 prior to stimulation with 10% foetal bovine serum. pERK1/2 expression was assessed by western blotting with calnexin as a loading control (1a). CI-1040 treatment leads to a dose-dependent reduction in pERK1/2 expression as a percentage of control (n = 3). Cell proliferation as assessed by BrdU ELISA (1b) shows CI-1040 at doses between 100nM and 10,000nM has no significant effect on cell proliferation (closed circles). Viability assays (1b, closed triangles) determined CI-1040 was cytotoxic at doses higher than 10,00nM (assay performed 3 times in triplicate. V to refers vehicle only. + refers to FBS-stimulated cells and–refers to non-stimulated cells.

To evaluate the *in vitro* efficacy of CI-1040 in rat fibroblasts, western blotting as shown in Fig 1A revealed a significant dose-dependent reduction in phospho- ERK1/2 (pERK1/2) expression with an IC50 of 5.8nM. There was a 70% reduction in pERK1/2 expression with a concentration of 10nM CI-1040 and complete inhibition at a concentration of 100nM. At a dose of 100nM, CI-1040 inhibited cell proliferation by 20% when compared to controls as measured by bromodeoxyuridine (BrdU) uptake but this was not statistically significant (Fig 1B). CI-1040 had no effect on cell cytotoxicity at doses between 100nM and 10μM but was cytotoxic at higher concentrations (Fig 1B).

### Phospho-ERK1/2 expression is upregulated after SNx and is inhibited *in vivo* by CI-1040

**Fig 2:**
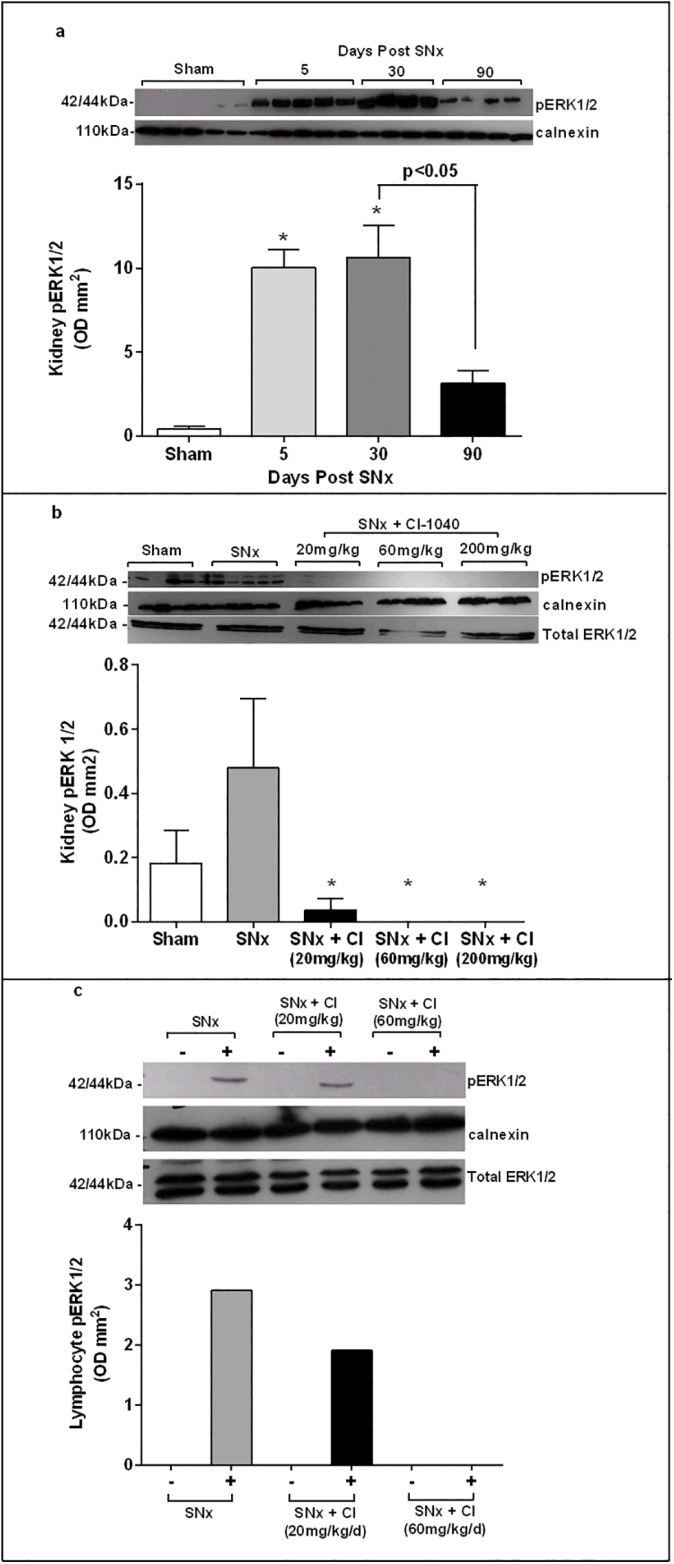
Phospho-ERK1/2 expression is increased after SNx and inhibited by CI-1040. SNx was performed and western blotting demonstrated (**a**) pERK1/2 expression in homogenates of remnant kidneys was significantly increased at days 5 and 30 with some decline at day 90 (n = 4–5 for each time point). * p<0.05 compared to sham- operated controls. (**b**) 5 days after SNx, CI-1040 60mg/kg/day inhibited pERK1/2 expression in remnant kidneys (n = 5, * p<0.05 compared to SNx controls) and in (**c**) PMA- stimulated (+) lymphocytes extracted from pooled blood samples. There was no pERK1/2 expression in unstimulated (-) lymphocytes. Densitometry values were corrected for protein loading using calnexin and total ERK1/2.

A chronic dosing strategy was determined by preliminary experiments involving a time course of pERK1/2 expression in the remnant kidneys of SNx rats and a 5-day acute dosing study to determine the concentration of CI-1040 required to inhibit pERK1/2 *in vivo*. As seen in Fig 2A there was minimal expression of pERK1/2 in kidneys of sham-operated animals at day 5 in comparison to a 23-fold increase in pERK1/2 expression observed in SNx kidneys at the same time point (p≤0.003). This level of activity persisted at day 30 but fell significantly at 90 days post-SNx (p≤0.03) although it remained elevated when compared to sham-operated controls. The efficacy of CI-1040 (20–200mg/kg/day) in inhibiting pERK1/2 expression *in vivo* was determined using an acute model of SNx with a single-step surgical technique.

5 days after SNx, pERK1/2 expression was completely abolished by CI-1040 at doses 60mg/kg/day and above (p<0.05) (Fig 2B). The lower dose of 20mg/kg/day, inhibited kidney pERK1/2 expression by 92% (p<0.05) but had no effect on lymphocyte pERK1/2 expression after PMA stimulation (Fig 2C). On the basis of this data a dose of 60mg/kg/day of CI-1040 was chosen for the chronic SNx study as it prevented ERK1/2 activation both in the kidney and in circulating lymphocytes. Administration was 30mg/kg b.i.d following toxicity assessments (% body weight loss and diarrhoea). This dosing schedule was also well tolerated in a phase 1 study in patients with cancer which demonstrated that twice daily dosing was the optimal dosing frequency for *in vivo* suppression of MEK [21].

### CI-1040 has no effect on blood pressure, albuminuria or kidney function after SNx despite complete inhibition of ERK1/2 activation

**Fig 3:**
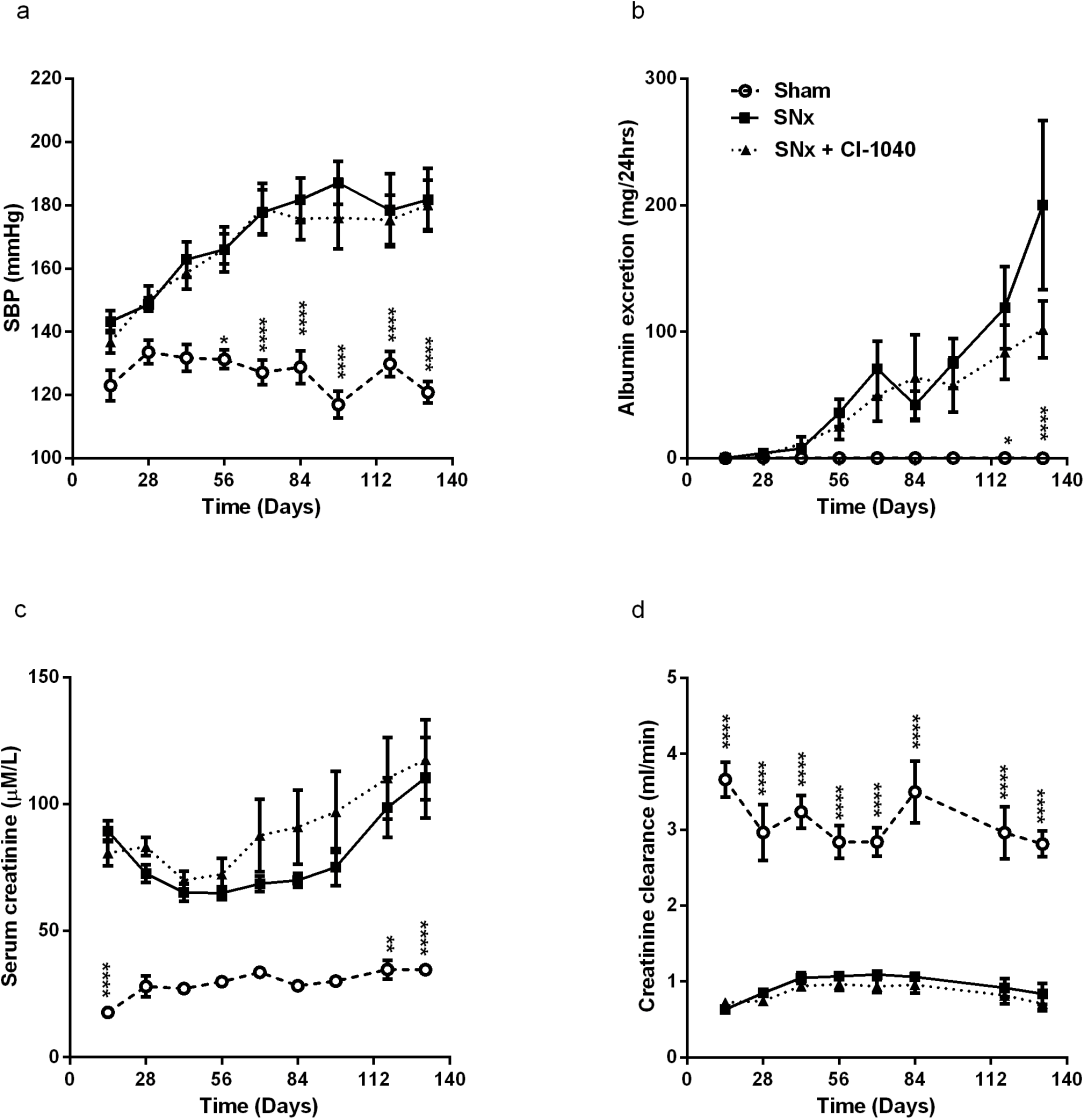
CI-1040 has no effect on blood pressure, kidney function and albuminuria after SNx. CI-1040 does not improve kidney function in the SNx rat; systolic blood pressure (SBP) (**a**), urinary albumin excretion (**b**), serum creatinine (**c**) and creatinine clearance (**d**) after 18 weeks treatment with either CI-1040 60mg/kg/d (closed triangles) or vehicle (closed circles) (n = 11 per group). Vertical bars indicate ± SEM, * p<0.05, ** p<0.001, *** p<0.0001 vs sham controls (open circles).

**Fig 4:**
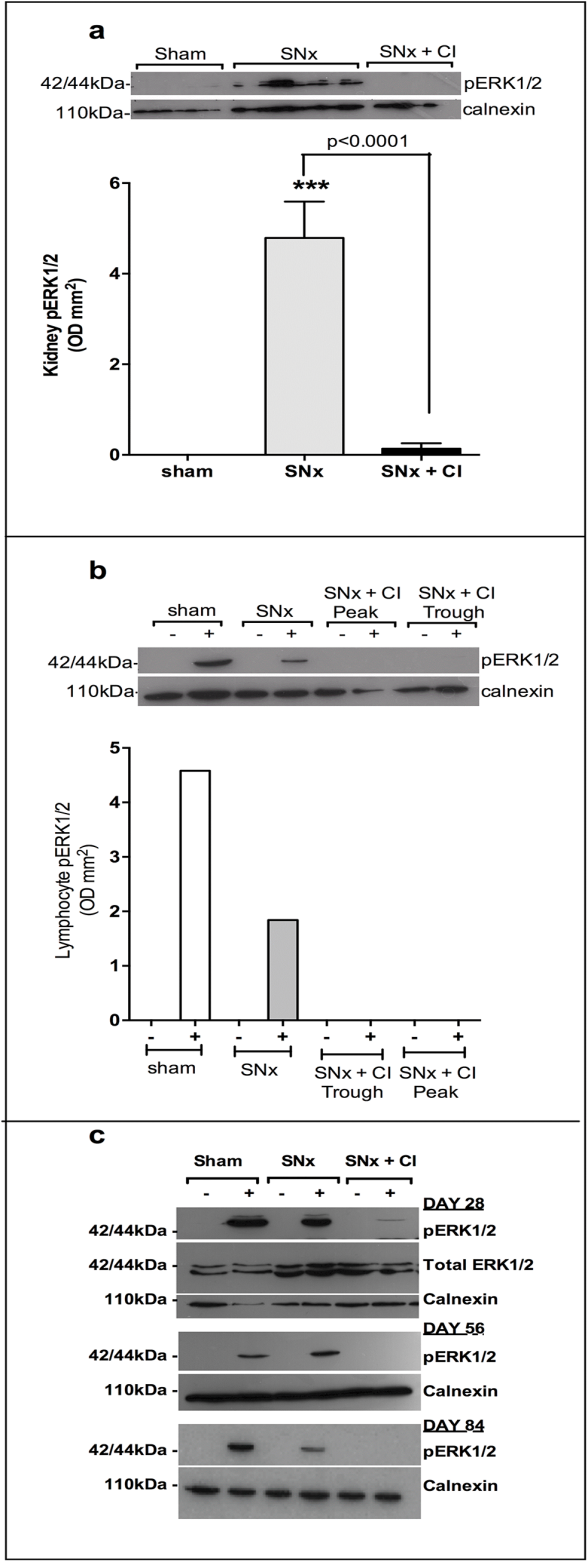
CI-1040 inhibits both renal and systemic pERK1/2 expression. CI-1040 abolished pERK1/2 expression in renal homogenates following SNx (**a**). Vertical bars indicate ± SEM, *** p<0.0001 vs sham controls. Pharmacological exposure was demonstrated between doses systemically by inhibition of pERK1/2 expression from PMA-stimulated (+) lymphocytes. There is no pERK1/2 expression in unstimulated (-) lymphocytes (**b**). Lymphocytes were harvested prior to dosing (trough) and 30 minutes after dosing (peak). Serial harvesting of lymphocytes every 14 days demonstrated complete inhibition of pERK1/2 expression in PMA stimulated (+) lymphocytes throughout the time course of the experiment Example blots of peak lymphocyte activity at days 28, 56 and 84 (c). D = day.

To ascertain the effect of MEK inhibition on kidney function, rats were subjected to a 2-step SNx with drug dosing commencing two days prior to surgery. Serum creatinine, albuminuria, creatinine clearance and blood pressure were measured on a fortnightly basis until sacrifice at day 131. By the end of the study, SNx controls were significantly hypertensive compared to sham operated controls (p<0.0001), albumin excretion had increased significantly (p<0.0001) and creatinine clearance had declined by 70% compared to sham operated animals (p<0.0001) (Fig 3A–3D). CI-1040 had no effect on blood pressure, albuminuria, serum creatinine or creatinine clearance. Terminal renal homogenates were examined for pERK1/2 expression (Fig 4A). There was a significant increase in pERK1/2 expression in SNx kidneys compared to sham controls (p<0.0001), which was almost completely abolished in SNx rats treated with CI-1040 (p<0.0001). In order to ensure that there was no ‘escape’ from MEK inhibition, throughout the duration of the study lymphocytes were harvested on a fortnightly basis and pERK1/2 expression assayed by western blot. A representative blot (Fig 4B) from terminal samples demonstrated full pharmacological coverage and inhibition of ERK1/2 activation between doses. Furthermore, there was complete pharmacological inhibition of pERK1/2 expression in PMA-stimulated lymphocytes for the duration of the study (Fig 4C) indicating lack of ‘escape’ by the development of resistance. Taken together, these data show that despite abolishing pERK1/2 activity in serum and in kidney tissue, CI-1040 had no impact on any parameter of kidney function.

### CI-1040 has no effect on fibrosis or myofibroblast number after SNx

**Fig 5:**
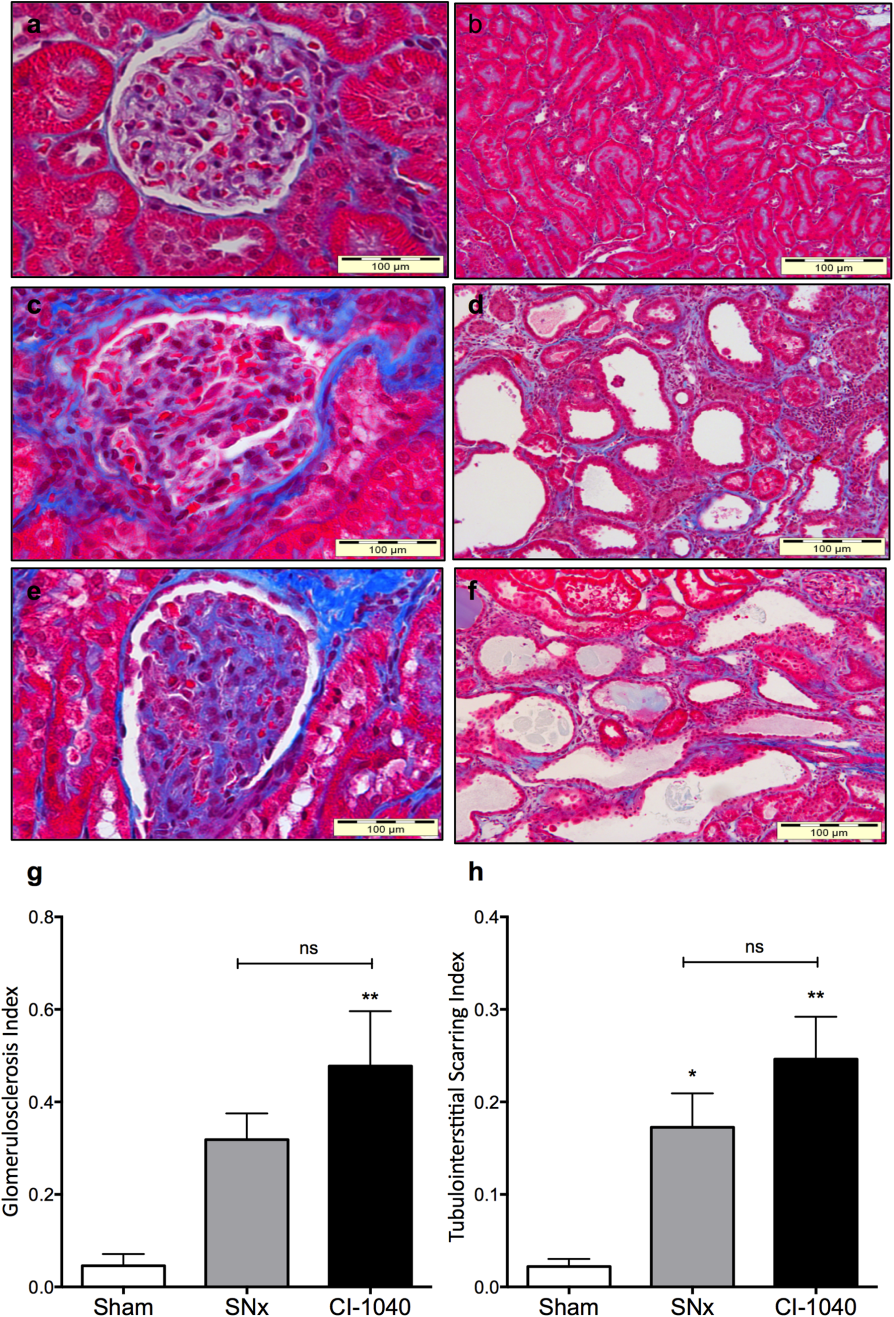
Glomerulosclerosis and tubulointerstitial fibrosis after treatment with CI-1040. Representative sections of glomeruli (400x) from terminal kidney tissue (131 days) stained with Masson’s trichrome obtained from (**a**) sham (**c**) SNx and (**e**) SNx plus CI-1040 60mg/kg/day rats. Group data are quantified in (**g**). Representative sections of the tubulointerstitium (200x) from terminal kidney tissue (131 days) stained with Masson’s trichrome obtained from (**b**) sham (**d**) SNx and (**f**) SNx plus CI-1040 60mg/kg/day rats. Group data are quantified in (**h**). Bars represent mean ± SEM, n = 7–11 per group* p<0.05, ** p<0.001 vs sham controls.

**Fig 6:**
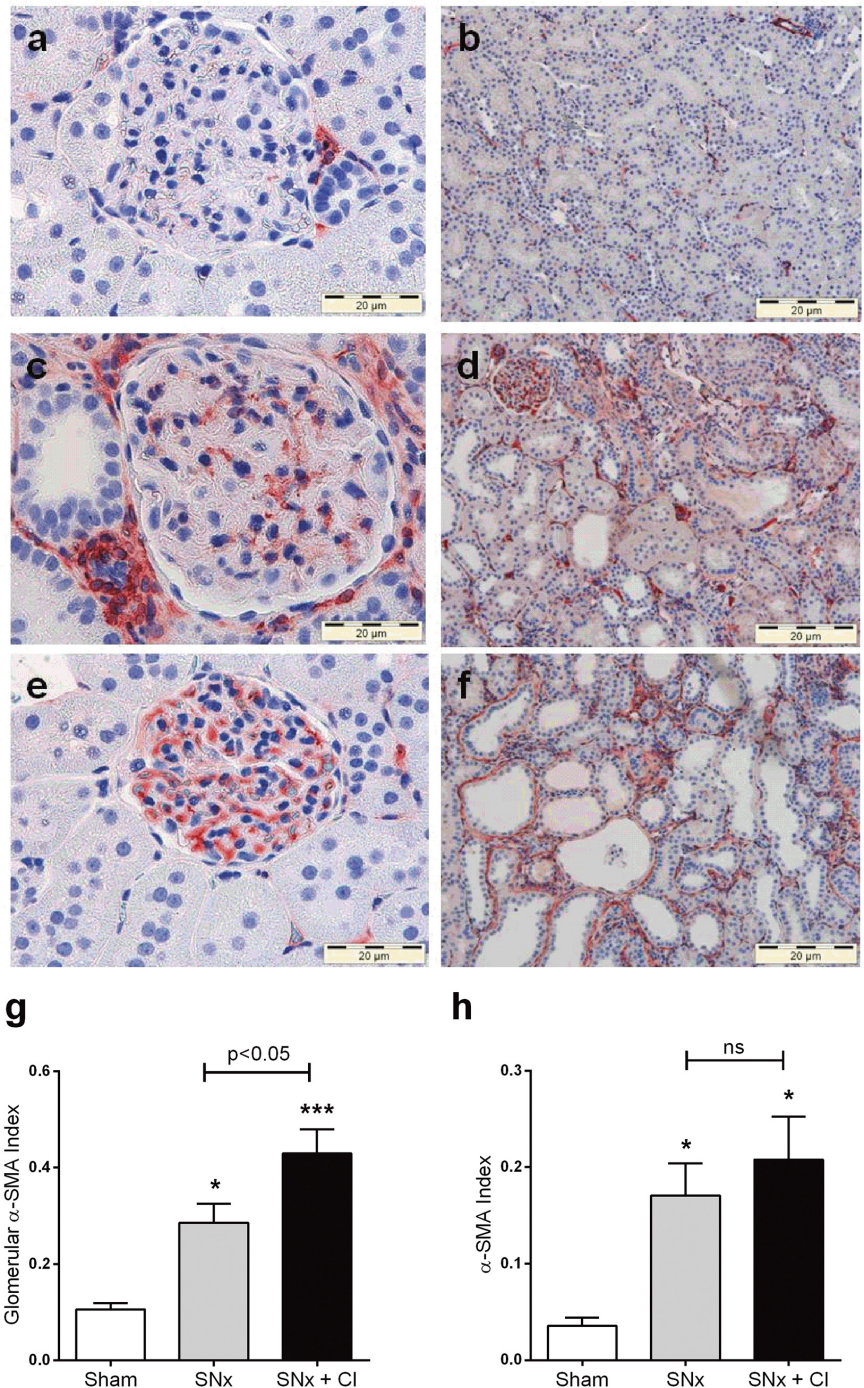
Myofibroblast number after treatment with CI-1040. Myofibroblast number as indicated by α-SMA staining. Representative sections of glomeruli (400x) from terminal kidney tissue (90 day) stained with α-SMA obtained from (**a**) sham (**c**) SNx and (**e**) SNx plus CI-1040 60mg/kg/day rats. Group data are quantified in (**g**). Representative sections of the tubulointerstitium (200x) from terminal kidney tissue (90 day) stained with α-SMA obtained from (**b**) sham (**d**) SNx and (**f**) SNx plus CI-1040 60mg/kg/day rats. Group data are quantified in (**h**). Bars represent mean ± SEM, n = 7–11 per group. * p<0.05, *** p<0.001 vs sham controls.

To quantitatively evaluate the impact of MEK inhibition on fibrosis, Masson’s Trichrome-stained kidney sections at sacrifice were subjected to multi-phase image analysis. At sacrifice there was a 16-fold and 8-fold (p<0.05) increase in glomerulosclerosis and tubulointerstitial fibrosis respectively after SNx although CI-1040 had no impact on fibrosis in either the glomerular or tubulointerstitial compartment (Fig 5A–5H). To determine whether CI-1040 could reduce the pool of myofibroblasts in the kidney either via effects on cell proliferation or EMT, α-SMA staining was performed on kidney sections at sacrifice. SNx led to a significant increase in myofibroblast accumulation in both glomerular and tubulointerstitial compartments (p<0.05) as determined by α-SMA staining, which was not inhibited by CI-1040 (Fig 6A–6H). Interestingly, CI-1040 actually increased α-SMA expression in the glomeruli (Fig 6G) but not in the tubulointerstitial compartment (Fig 6H).

### Inhibition of ERK1/2 activation leads to compensatory activation of p38, cJun and increased expression of PAI-1 after SNx

**Fig 7:**
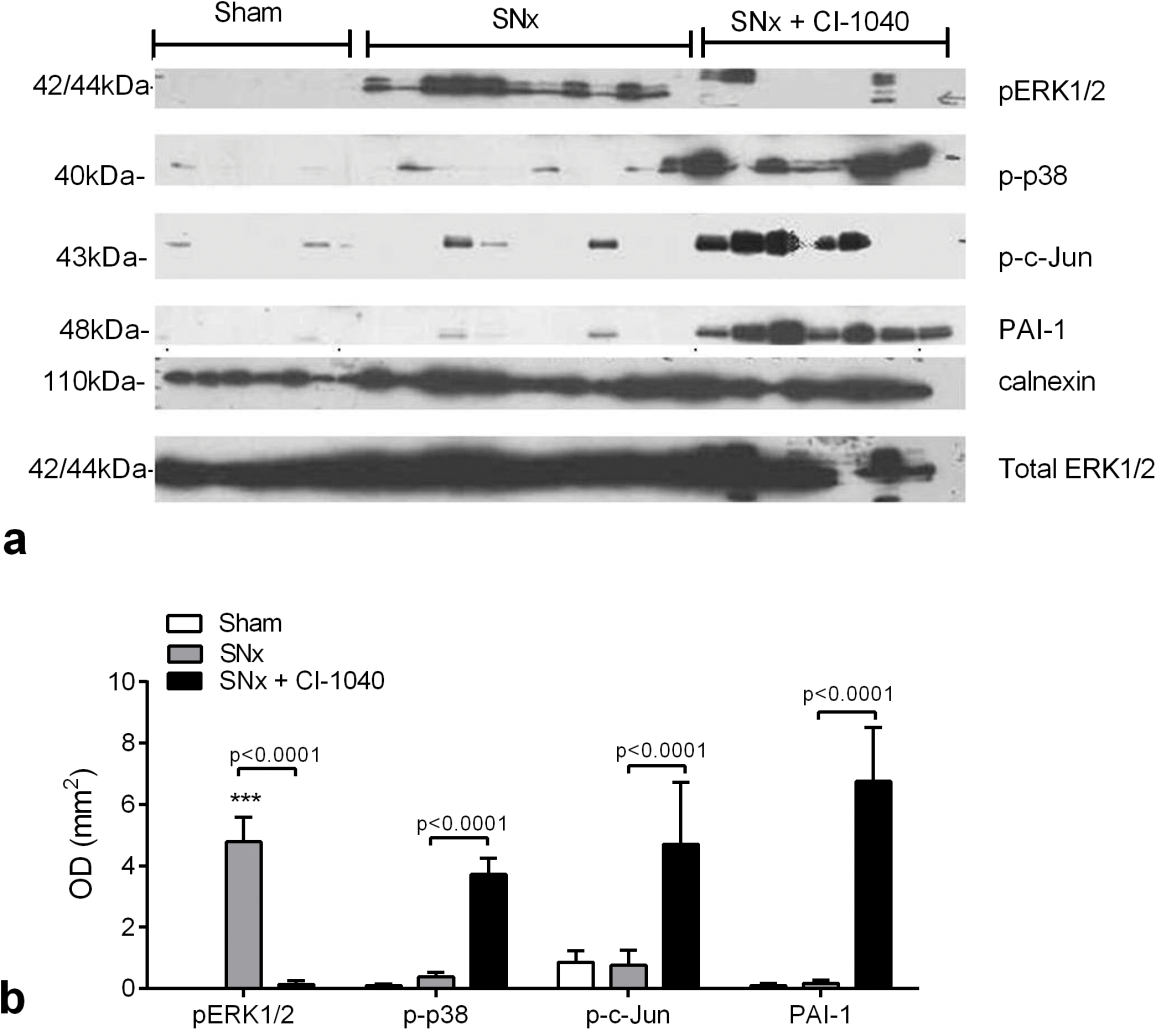
CI-1040 inhibits phospho-ERK1/2 expression after SNx and upregulates p38, cJun and PAI-1. (**a**) terminal kidney homogenates were western blotted for pERK1/2, phospho-p38 (p-p38), phospho-cJun (p-c-Jun) and PAI-1 with calnexin and total ERK1/2 acting as loading controls. (**b**) Densitometry values plotted with data representing mean +/- SEM, n = 6–11 per group. Statistical significance determined by one-way ANOVA. ***p<0.0001.

As increased ERK1/2 activation has previously been shown to be associated with kidney fibrosis we investigated whether the lack of effect of inhibiting pERK1/2 *in vivo* was a result of compensatory upregulation of other MAPK signalling pathways. Western blotting of terminal kidney homogenates revealed significant increased expression of pERK1/2 after SNx (p<0.001) with almost complete inhibition pERK1/2 expression after treatment with CI-1040 (p<0.001) (Fig 7A and 7B). There was minimal phospho-p38 (p-p38), phospho-cJun ((p-cJun), a marker of JNK activity) and plasminogen activator inhibitor 1 (PAI-) expression after SNx. Treatment with CI-1040 resulted in significant increases in the activation of p38 (p<0.001), cJun (p<0.001) and expression of PAI-1 (p<0.001) when compared to untreated SNx kidneys (Fig 7A and 7B) which inversely correlated with pERK1/2 expression (PAI-1, p<0.05; p-cJun, p<0.05).

## Discussion

The key finding of this study is that MEK inhibition with CI-1040 after SNx results in compensatory upregulation of other MAPKs such as p38, JNK (as shown by increased p-cJun activity) as well as increased expression of PAI-1, which is well characterised as having both pro-fibrotic and anti-fibrinolytic properties [24]. However CI-1040 has no impact on any biochemical (creatinine clearance, albuminuria) or histological marker of kidney injury after SNx despite complete inhibition of pERK1/2 expression both in kidney tissue and in lymphocytes. Kidney fibrosis following SNx is associated with ERK1/2 activation with little activation of other MAPKs such as p38 and JNK. However, inhibiting ERK1/2 activation simply leads to the upregulation and utilisation of ERK-independent pathways such as p38 and JNK. The twice daily dosing regime of CI-1040 (60mg/kg/day) used in our study is broadly comparable to previous *in vivo* studies [22] and to the higher doses of the drug that have been used in phase 1 clinical studies [21]. In contrast to the clinical trial the rats in this study did not suffer adverse effects at this higher dosage.

Increased ERK1/2 activation in association with fibrosis has been observed in human kidney biopsies and recent data in IgA nephropathy indicates that pERK1/2 expression was associated in those with significant proteinuria [19, 25]. Furthermore IgA1-dependent ERK1/2 activation controlled the secretion of pro-inflammatory cytokines by human mesangial cells [25]. In addition ERK1/2 is activated in a variety of *in vivo* models of kidney disease including Thy-1.1 mesangioproliferative glomerulonephritis, crescentic glomerulonephritis and in unilateral ureteric obstruction (UUO) [4, 26, 27]. However, the impact of pERK1/2 inhibition has been variable. In mouse models of polycystic kidney disease pERK1/2 has been shown to improve kidney function and inhibit cell proliferation [28, 29] and in a model of chronic allograft nephropathy the effects of CI-1040 on attenuating fibrosis appeared to be mediated by a reduction in TGF-β1 biosynthesis in the allograft [22]. There are similar data in models of cisplatin-induced renal injury and ischemia-reperfusion injury where targeting of Ras-dependent signalling and pERK1/2 inhibition attenuates kidney damage [30, 31]. In contrast, in the UUO model pERK1/2 inhibition reduced tubular cell proliferation and accumulation of inflammatory cells but had no impact on kidney fibrosis [27]. Similarly in the Heymann nephritis model of membranous nephropathy inhibiting pERK1/2 worsened DNA damage in podocytes [32] suggesting that in different contexts activation of ERK1/2 may be an appropriate rather than a maladaptive response to injury.

The development of fibrosis after SNx results initially from compensatory renal growth due predominantly to hypertrophy of tubular epithelial cells followed by upregulation of key pro-fibrotic growth factors such as TGF-β1 leading to excessive production of ECM and subsequent fibrosis [33]. Myofibroblasts are key drivers of fibrosis and the myofibroblast pool in the tubulointerstitium derives from a variety of sources including activation and proliferation of resident interstitial fibroblasts, activation of pericytes, tubular EMT and endothelial mesenchymal transition [34]. ERK1/2 mediates both cell hypertrophy, TGF-β1-mediated ECM production as well as myofibroblast activation and proliferation and thus seemed a logical target in progressive, fibrotic kidney disease [35].

We demonstrated that despite inhibiting pERK1/2 phosphorylation *in vitro* and *in vivo*, CI-1040 had no significant impact on fibrosis and myofibroblast number after SNx. We did not assess the effects on other organs as we were primarily interested in the glomerular and tubulointerstitial effects of CI-1040 as tubulointerstitial fibrosis has long being recognised as the best histological predictor of progressive kidney disease. Interestingly we observed that CI-1040 appeared to actually increase fibrosis of the glomeruli although this was not observed in the tubulointerstitial compartment. There is no clear explanation for this disparity but it does imply that increased ERK1/2 activation in the glomeruli is an appropriate response to tissue injury and thus inhibition of ERK1/2 may actually impede tissue repair. Alternatively, localisation of α-SMA staining to the glomeruli might reflect de novo expression by mesangial cells rather than myofibroblasts. Although our data demonstrates ‘global’ inhibition of ERK1/2 by CI-1040 we cannot be sure whether ERK1/2 was completely inhibited in myfibroblasts as the overall number in kidney homogenates may be comparatively small to other cell types present. Immunofluorescence (IF) of tissue sections with phospho-ERK1/2 would have helped localise the cellular compartments of MEK inhibition but we were unable to achieve IF of sufficient quality to enable quantification of tissue expression of p-ERK1/2. We did not investigate other tissues for MEK activity–however rats treated with CI-1040 appeared to tolerate it without problems and in particular there was no observed diarrhoea which one might anticipate given the labile cell population in the gastrointestinal tract.

A particular strength of our study was the demonstration that both kidney and lymphocyte pERK1/2 expression were downregulated indicating both systemic and renal inhibition of ERK1/2 activation. There are a number of possible reasons why, despite almost complete knockdown of activated ERK1/2, fibrosis was not inhibited. Firstly it is clear that ERK is not always required for cell proliferation [36] and secondly blocking MEK may simply lead to compensatory signalling through parallel MAPK pathways such as p38 and JNK, which can drive proliferation and fibrosis. Indeed we demonstrated that reduced expression of pERK1/2 led to increased activation of both JNK and p38 and it is likely that this accounts for part of the reason why ERK1/2 inhibition had no impact on fibrosis. Both p38 and JNK pathways are implicated in a variety of kidney diseases through effects on inflammation, fibrosis and tubular damage [37, 38]. Immunostaining of human renal biopsies demonstrates correlation of p38 and JNK activation with fibrosis and inflammation in a number of glomerular and tubulointerstitial diseases [37, 39]. JNK blockade in an experimental model of anti-GBM glomerulonephritis attenuated both glomerular and tubulointerstitial damage with the effect being mediated by a reduction in the release of pro-inflammatory mediators such as TNF-alpha from macrophages [40]. Inhibition of p38 again attenuated injury in a crescentic nephritis model mediated via reduced infiltration of macrophages and neutrophils [41]. In a Heymann nephritis model p38 inhibition exacerbated proteinuria with evidence of augmented complement- mediated cytotoxicity [42] whilst Ohashi and colleagues demonstrated that a p38 inhibitor exacerbated renal injury and fibrosis in SNx due to upregulation of activated ERK1/2 [43]. This suggests that *in vivo* the potential therapeutic benefit of targeting a single MAPK pathway can be circumvented by upregulation of alternate MAPK pathways. Although CI-1040 is regarded as a highly selective ATP non-competitive MEK1/2 inhibitor, 100-fold more selective than MEK5 [44], this study cannot rule out the possibility that activation of p38 and JNK is a result of generic ERK1/2 inhibition or an off target effect of CI-1040. Further work with other MEK inhibitors would be required for clarification. Furthermore it is possible that these effects of CI-1040 maybe mediated by effects on inflammation but we did not study this in detail as SNx is not primarily an inflammatory model of kidney disease but exploratory experiments using cytometric bead arrays were inconclusive in terms of effects on pro-and anti-inflammatory cytokines.

We also demonstrated that PAI-1 expression is increased after administration with CI-1040. Urokinase-type/tissue-type plasminogen activator (uPA/tPA) and plasmin promote proteolytic degradation of ECM proteins, with their activity being inhibited by PAI-1 [45, 46]. Increased PAI-1 can promote fibrosis not only by inhibiting the breakdown of ECM but also by stimulating recruitment of interstitial macrophages and promoting increased expression of profibrotic genes [45]. Interestingly both p38 and JNK have been shown to upregulate PAI-1 expression in fibroblasts [46] and this may be another mechanism by which increased JNK and p38 activity promotes fibrosis.

In summary we have shown that ERK1/2 is activated after SNx with little activation of JNK and p38 and minimal expression of PAI-1. Inhibiting ERK1/2 activation with a MEK inhibitor had no effect on kidney fibrosis but was associated with a striking upregulation in p38 and JNK signalling and increased expression of PAI-1. Taken together this suggests that there is significant crosstalk and redundancy between parallel MAPK signalling pathways that underpin fibrosis after SNx and so targeting single, downstream kinases such as ERK1/2 is unlikely to be an effective therapeutic strategy in kidney fibrosis.

## Materials and Methods

All procedures were carried out under license according to regulations laid down by Her Majesty’s Government, United Kingdom (Animals Scientific Procedures Act, 1986). All procedures were carried out at The University of Sheffield with approval by Animal Welfare & Ethical Review Body (ASPA Ethical Review Process).

### MEK Inhibitor CI-1040

CI-1040 was provided by Pfizer Ltd, UK. Stock solutions were prepared in dimethyl sulphoxide (DMSO). For *in vivo* studies, CI-1040 was diluted in 5% (v/v) DMSO, 5% cremophor EL (Sigma Aldrich, UK) in saline.

### *In vitro* Studies

Normal rat kidney interstitial fibroblast cells (NRK49F) were obtained from American Type Culture Collection (Manassas, VA, USA). Cells were cultured in DMEM supplemented with 10% FCS and 5% antibiotics (Invitrogen, Paisley, UK) and maintained at 37°C in a humidified 5% CO2 incubator. 70% confluent monolayers grown on petri dishes were synchronised by serum starvation for 24 hours (negative control) prior to stimulation for 5 minutes with serum-supplemented medium (positive control). CI-1040 (1–1000nM) was added to medium overnight during serum starvation. ERK activation was measured by phospho-ERK1/2 (pERK1/2) expression. Cells were washed twice with ice-cold PBS containing 1mM sodium vanadate, RIPA lysis buffer (+ protein and phosphatase inhibitors) was added to the plates and the cells were scraped. Pharmacological activity of CI-1040 was determined in cell lysates by a western blot for pERK1/2. *In vitro* sensitivity to CI-1040 was assessed by a cytotoxicity assay (CCK-8 assay, Sigma Aldrich, UK) and proliferation was measured with a BrdU ELISA (Roche, UK) as per the manufacturer’s instructions following 24 hour incubation with CI-1040 (0.1–100μM) in the presence of 10% FBS.

### Experimental animals and protocol

Male Wistar Han rats (200–250g, Harlan, Bicester, UK) were housed 4 to a cage at 20–22°C and 45% humidity on a 12-h light/dark cycle and allowed free access to standard rat chow (protein/casein content 18%) and tap water. Animals were subjected to two-step sub-total nephrectomy (SNx) under isofluorane anaesthesia (n = 22). Briefly, upper and lower poles of the left kidney were excised surgically followed by right uni-nephrectomy 7 days later. Control rats were subjected to sham operation in the absence of kidney manipulation (n = 7).

SNx rats were divided into two groups: SNx + vehicle (intraperitoneal (i.p) injection, twice daily, n = 11) and SNx + 60mg/kg/day CI-1040 given in divided doses, twice daily as an i.p. injection (n = 11). Renal function was assessed fortnightly by the collection of urine (24hr metabolic cages) and blood samples. Urine was analysed for albumin by ELISA (Bethyl Laboratories, Cambridge Bioscience, UK). Serum and urine were analysed for creatinine using a Jaffe kinetic reaction (Department of Clinical Chemistry, Royal Hallamshire Hospital, Sheffield Teaching Hospitals NHS Trust). Although this colorimetric assay has been criticized for overestimation (due to cross-reactions with bilirubin, glucose, hemoglobin) the Jaffe reaction remains the cornerstone of most current routine methods [47]. Rats were acclimatised to restraining cages prior to blood pressure measurements by computerised tail-cuff plethysmography (IITC Life Science, Woodland Hills, California, USA). All functional assessments were performed at the same time of day throughout the experimental period.

The systemic pharmacological activity of CI-1040 was monitored at regular intervals throughout the course of the study by collecting blood samples prior to and 30 minutes after CI-1040 injection. The timing of the samples was taken to represent peak and trough drug activity. Samples from each treatment group were pooled then divided in two with and without 5 minutes phorbol myrisate acetate (PMA) stimulation prior to extraction of the lymphocytes by Ficoll separation. Lymphocyte lysates underwent a western blot for pERK1/2 expression.

### Tissue Analysis

Renal cortical tissue was divided into quarters for analysis. One quarter was fixed in 10% neutral buffered formalin, paraffin-embedded, sections cut (4μm) and stained with Masson’s trichrome (Sigma Aldrich, UK) as per manufacturer’s instructions. Unstained sections were dehydrated before undergoing heat-induced epitope retrieval using basic tissue unmasking fluid (TUF, Zymed Labs). Myofibroblast accumulation was assessed by incubation with rabbit polyclonal α-smooth muscle actin (α-SMA) antibody (1:100, DAKO), goat anti-rabbit HRP conjugated secondary antibody (1:200, DAKO) and AEC visualisation (Vector Labs, UK). Transverse sections of the kidney, which passed through the papilla, were selected. The sections were viewed down a light microscope with a x40 (glomerular) or x20 (tubulointerstitium) flat field objective. Data were collected from 1 section per kidney whereby a minimum of 10 glomeruli or a series of adjacent fields extending perpendicularly from the cortex to the junction between the outer and the inner stripes of the outer medulla were acquired using a CC12 digital camera (Soft Imaging Systems, Germany).

Staining for α-SMA and Masson’s Trichrome was assessed using three-phase image analysis (Analysis 3.2 software, Soft Imaging Systems, Germany) ensuring total phase coverage greater than 95%. Cytoplasmic staining for α-SMA (red) was expressed as a ratio of the nuclear stain (blue). For Masson’s Trichrome, staining for ECM protein (blue) was expressed as a ratio of the cellular stain (red) thus correcting for cell number.

### Western Blots

The remaining renal tissue was snap frozen in liquid nitrogen. Kidney samples were homogenised in RIPA buffer for protein analysis by western blotting. Protein concentration was measured before electrophoresing on 12% (w/v) SDS- polyacrylamide gels. After electrotransfer to Immobilon-P membranes (Millipore, USA), membranes were blocked at room temperature with 10% (w/v) milk powder for 1 hour. The primary antibodies against pERK1/2 (1:1000, Cell Signalling, USA), total ERK1/2 (1:1000, Cell Signalling), calnexin (1:1000, Sigma Aldrich, UK), phospho-cJun(p-cjun) (1:1000, Cell Signalling), phospho-p38(p-p38) (1:1000, Cell Signalling) and PAI-1 (1:1000, Abcam, UK) were incubated overnight at 4°C. After washing, the membranes were incubated with goat IgG-horseradish peroxidise conjugated secondary antibodies (DAKO) at a final dilution of 1:20,000 for 1 hour at room temperature. After washing, antibody binding was visualised with enhanced chemiluminescence (Roche, UK) and autoradiography. The expression levels of proteins were analysed on western blots by using 20μg of total cell lysates with loading errors corrected for using calnexin due to its different molecular weight.

### Statistical analysis

Data are expressed as mean ± SEM. Functional data were analysed by two-way analysis of variance (ANOVA), followed by Bonferroni’s multiple comparison post hoc test. Histological and western blot results were analysed by one-way ANOVA. P<0.05 was considered statistically significant.
